# Comparative Diagnostic Efficacy of Microscopy, LAMP and PCR for Detection of Bovine Babesiosis in New Valley Governorate, Egypt

**DOI:** 10.1007/s11686-025-01194-w

**Published:** 2026-01-13

**Authors:** Khatib H. Abdelwahab, Mohammed E. M. Tolba, W Senosy, Ahmed M. Ahmed, Abeer A. Khedr, Khaled Abdelaziz, Shawky M Aboelhadid, Wafaa G. Mahmoud

**Affiliations:** 1 Department of Parasitology, Faculty of Veterinary Medicine, New Valley University, New Valley, El-Khargah, 72511 Egypt; 2 Department of Clinical Microbiology and Parasitology, College of Medicine, King Khalid University, Abha, Saudi Arabia; 3 Department of Theriogenology, Faculty of Veterinary Medicine, New Valley University, El- Kharga, 72511 Egypt; 4 Department of Animal Medicine (infectious diseases), Faculty of Veterinary Medicine, New Valley University, El-Khargah, 72511 Egypt; 5 Department of Biosciences, Durham University, Durham, UK; 6 Department of Animal and Veterinary Science, Clemson University, Clemson, SC 29634 USA; 7 Clemson University School of Health Research (CUSHR), Clemson, SC 29634 USA; 8 Department of Parasitology, Faculty of Veterinary Medicine, Beni-Suef University, Beni- Suef, 52611 Egypt

**Keywords:** Bovine babesiosis, Cattle, *Babesia*, LAMP, PCR

## Abstract

**Purpose:**

Bovine babesiosis poses a serious threat to cattle in tropical and subtropical regions. This study compared 3 methods to detect babesiosis in naturally infected cattle; microscopy, loop-mediated isothermal amplification (LAMP), and conventional PCR (cPCR), in Egypt’s New Valley Governorate.

**Methods:**

A total of 339 tick-infested cattle were examined for *Babesia* infection. Microscopic examination was done. Genomic DNA extraction for the LAMP and PCR assays was carried out. LAMP was performed and compared with cPCR.

**Results:**

Molecular methods revealed higher infection rates than microscopic examination: LAMP detected *Babesia* DNA in 40.7% (138/339) of samples, cPCR in 41.9% (142/339), while microscopy identified 31.9% (108/339). Clinically infected cattle exhibited fever, hemoglobinuria, pallor, and weight loss. Seasonal variation showed peak prevalence in summer (37.8%) and the lowest in winter (20%). Based on comparative analysis, LAMP showed 97.18% sensitivity, 100% specificity, and almost perfect agreement with cPCR (κ = 0.98, *P* = 0.13).

**Conclusions:**

These findings demonstrate high *Babesia* prevalence in tick-infested cattle and underscore LAMP’s value as a rapid, sensitive, and field-friendly tool for surveillance.

## Introduction

Babesiosis is an intraerythrocytic protozoan disease caused by species of the genus *Babesia*, transmitted primarily by *Rhipicephalus (Boophilus) ticks* [1]. The most notable species associated with cattle infections are *Babesia bigemina and Babesia bovis*, which are widespread across tropical and subtropical regions, including Africa, Asia, Australia, the Americas, and southern Europe [2]. These infections result in substantial economic losses in livestock production, with annual global losses estimated between USD 13 and 19 billion [3].

In Egypt and other affected regions, the disease is linked to significant reductions in milk and meat yields, as well as increased morbidity and mortality among cattle [4, 5]. Recent studies have documented the distribution and prevalence of *Babesia* species in various geographical areas, highlighting the widespread occurrence of *B. bigemina and B. bovis* among cattle populations in Africa and the Middle East [6, 7].

While *B. bigemina* exhibits a broader geographical distribution, *B. bovis* infections are more severe and often fatal due to complications such as hyperthermia, hemoglobinuria, anorexia, and neurological symptoms [1, 8]. The pathogenicity of *B. bovis* is greater than that of *B. bigemina*, as *B. bovis* infections are characterized by sequestration of infected erythrocytes in the microcapillaries of vital organs, such as the lungs, kidneys, and brain [9]. This process can result in severe organ dysfunction and systemic shock, leading to fatal outcomes. Moreover, cattle recovering from *B. bovis* infections frequently serve as chronic carriers, facilitating the continuous spread of the parasite through tick vectors [2, 7].

Traditional diagnostic methods for bovine babesiosis, such as Giemsa-stained blood microscopy, have low sensitivity, particularly in subclinical or latent infections [6]. Molecular diagnostics, such as conventional polymerase chain reaction (PCR), have demonstrated higher sensitivity and specificity in detecting *Babesia* sp. during early infection stages [8, 9]. However, PCR is limited by its cost and need for specialized equipment, making it less accessible in resource-limited settings [7].

More recently, quantitative PCR (qPCR) assays have been developed and validated for the accurate detection and differentiation of *B. bigemina and B. bovis* in cattle samples, offering enhanced diagnostic precision [8, 9]. Other immunological methods, including indirect fluorescent antibody tests (IFAT) and enzyme-linked immunosorbent assays (ELISA), have also proven effective in diagnosing chronic infections and field surveys in bovines [3–5].

More recently, loop-mediated isothermal amplification (LAMP) has emerged as a promising diagnostic tool for *Babesia* infections. LAMP offers rapid, sensitive, and specific detection under isothermal conditions, eliminating the need for complex equipment. More importantly, it enables visual detection of *Babesia* genomic material in a sample, making it practical for field applications [10]. LAMP has been applied successfully to diagnose parasitic diseases in both humans, such as *Leishmania* and *Plasmodium*, and animals, including *Babesia* [11]. Thus, the development of *Babesi*a species-specific LAMP assay targeting *B. bigemina* and *B. bovis* provides a useful tool for the clinical detection of *Babesia* infection, particularly in countries where bovine babesiosis is endemic [12].

In this study, a LAMP assay targeting the 18 S rRNA gene of *B. bigemina and B. bovis* was optimized for the detecting of babesiosis in naturally infected cattle, and its diagnostic performance was evaluated in comparison with conventional microscopy and PCR to determine its sensitivity and specificity.

## Materials and Methods

### Ethical Approval

This study was approved by the Research Ethics Committee of the Faculty of Veterinary Medicine, New Valley University (Approval No. 02-2-7-2023-3). All procedures involving animals were conducted in accordance with institutional guidelines and local legislative requirements.

### Study Area

This study was conducted in the New Valley Governorate, Egypt—a region characterized by an arid climate and a landscape dominated by deserts and oases. The New Valley was selected for this study as cattle breeding constitutes one of the main agricultural activities in this region, and previous studies have documented tick infestations [13], and the occurrence of tick-borne diseases (TBDs), including babesiosis. The study area is located approximately at 24°2′45.70″ N latitude and 27°9′44.91″ E longitude [14].

### Study Design and Sample Collection

The research, conducted between September 2023 and November 2024, aimed to detect *Babesia* species in cattle. A total of 339 cattle naturally infested with ticks were selected using simple random sampling. All samples were screened using the three diagnostic assays, and cattle were categorized as microscopy-positive or molecular-positive to differentiate between clinical and subclinical cases. The sample size was calculated based on the estimated prevalence of bovine babesiosis in similar regions (approximately 30%) and a 95% confidence level to ensure statistical significance [15]. The sampled animals were classified into three age groups: calves (under 1 year), young (1–3 years), and adults (over 3 years), with age determined through dentition patterns [16] and information provided by owners. Clinical data on fever, jaundice, and hemoglobinuria were recorded. Animals were sampled during three different seasons: Summer (June–August), Autumn (September–November), Winter (December–February), and Spring (March–May) [17]. Samples were collected from both field animals and those presented to local veterinary clinics in the study area.

### Blood Collection

Whole blood samples (5 mL) were collected from the jugular vein of each animal using two sterile EDTA vacutainer tubes (total 10 mL per animal). The use of two separate tubes ensured dedicated samples for immediate microscopic examination and molecular analysis, avoiding degradation from repeated freeze–thaw cycles. One tube was processed on the same day for parasitological examination, while the other was stored at − 80 °C for subsequent molecular analysis [18].

### Parasitological Microscopic Examination of Blood

Thin blood smears were prepared, air–dried, and fixed with methanol. Smears were stained with Giemsa and examined under a light microscope (Olympus BX43F, Tokyo, Japan). Images were captured using a camera (Olympus EP50, Tokyo, Japan) at the Photomicrograph Laboratory of the Parasitology Department, Faculty of Veterinary Medicine, New Valley University. Morphological features of *Babesia* spp. were recorded [19].

### Molecular Examination

#### Loop–Mediated Isothermal Amplification (LAMP) Assay

Positive and negative controls were represented by field samples that were previously confirmed to be positive or negative by PCR followed by sequencing of the amplified products for the related genes in the Animal Health Research Institute. Genomic DNA extraction for the LAMP assay was carried out using the QIAamp DNA Mini Kit (Catalogue No. 51304, Qiagen, Germany) according to the manufacturer’s instructions. The LAMP reaction was set up in a total reaction volume of 30 µL, containing 3 µL of 10× BSM buffer, 1 µL of BSM DNA polymerase (ThermoScientific, Germany), 1 µL each of the outer primers F3 and B3, 2 µL each of the inner primers FIP and BIP (20 pmol concentration), and 10 µL of nuclease-free water. The primers targeted the *Babesia* 18 S rRNA gene [20]. Amplification was performed at 90 °C for 40 min, followed by enzyme inactivation at 80 °C for 2 min. Positive samples turned green upon addition of SYBR Green I (MERCK, Germany), while negative samples remained orange. Results were confirmed by 1.5% agarose gel electrophoresis and were stained by 1% ethidium bromide.

####  LAMP Assay (Field Test)

Primers were used in a 30 µL total reaction volume containing 3 µL of 10× BSM buffer, 1 µL of BSM DNA polymerase (ThermoScientific, Germany), 1 µL each of primers (F3 and B3) and 2 µL of each primer (FIP and BIP) at 20 pmol, 10 µL of DNA template (approximately 100 ng), SYBR Green I dye (MERCK, Germany), and nuclease-free water to reach the total volume. To further confirm the results, LAMP products were analyzed through 1.5% agarose gel electrophoresis prepared with Tris Borate EDTA (TBE) buffer, and stained with ethidium bromide (10 mg/mL). To further confirm the results, LAMP products were analyzed through agarose gel electrophoresis using a 1.5% agarose gel prepared with Tris Borate EDTA (TBE) buffer and stained with ethidium bromide (10 mg/ml). Visualization of DNA bands was carried out under UV light using a gel documentation system (Alpha Innotech).

#### Conventional PCR (cPCR) Assay

Genomic DNA extraction for cPCR was carried out as in the LAMP assay. The assay targeted *Babesia* 18SrRNA gene, using genus-specific primers described by Abd–Elrahman et al. [21], which amplify a 240 bp product. The primers were selected for their broad detection range across the *Babesia* genus. *Babesia* PCR reactions were set up in a total volume of 25 µL, containing 12.5 µL of 1X Essential Amp PCR Master Mix, 7.5 µL of PCR-grade water, 1 µL of each primer (yielding a final concentration of 0.4 µM each), and 2 µL of template DNA (approximately 100 ng). Cycling conditions were: 94 °C for 2 min; 35 cycles of 94 °C for 30 s; 55 °C for 40 s; and 72 °C for 40 s; final extension at 72 °C for 10 min.

####  Evaluation of Diagnostic Techniques

A total of 20 blood samples collected from clinically suspected cattle were evaluated. Diagnostic performance of microscopy, LAMP, and cPCR was assessed using cPCR as a reference. Sensitivity, specificity, PPV, NPV, and CPV were calculated as in [20].

####  Data Analysis

Risk factors associated with *Babesia* infection in cattle were analysed using R software (version 4.2.2) and the epitools package (Team 2010). Descriptive statistics summarized infection prevalence across seasons (Summer, Autumn, Winter, Spring), age groups (< 1 year, 1–3 years, 3 years), gender (Male, Female), and study locations. Risk ratios (RR) and odds ratios (OR) with 95% confidence intervals (CI) were calculated to evaluate associations between each variable and infection status, using Summer, < 1 year, and Male as reference categories. Wald Chi-square tests were applied to determine statistical significance, with a p-value < 0.05 considered significant. The diagnostic accuracy of blood smear microscopy, LAMP, and (cPCR was assessed using cPCR as the reference method. Diagnostic metrics, including sensitivity, specificity, positive predictive value (PPV), negative predictive value (NPV), and combined predictive value (CPV), were computed based on true positive (TP), true negative (TN), false positive (FP), and false negative (FN) classifications. The formulas applied were, Sensitivity = TP / (TP + FN), Specificity = TN / (TN + FP), PPV = TP / (TP + FP), NPV = TN / (TN + FN), and CPV = (TP + TN) / (TP + FP + TN + FN). Agreement between tests and the reference standard was measured using the Kappa coefficient, with values closer to 1 indicating stronger concordance. Results were expressed as percentages and statistical indices for each diagnostic method.

## Results

### Clinical Observations of the Infected Cattle

Cattle suspected of babesiosis infection showed a range of clinical signs. Animals heavily infested with ticks showed lethargy, severe weight loss, increased respiratory rate, and hemoglobinuria. Pallor of the mucous membranes was also common. In addition, affected animals showed signs of systemic illness, including anemia and diarrhea. Pyretic responses were recorded, with body temperatures reaching up to 40.5 °C. Given that hemoglobinuria is a recognized clinical sign of babesiosis and can often be detected microscopically, conventional Giemsa-stained blood smears were also evaluated alongside the LAMP assay.

### Microscopic Identification of Blood Samples

**Fig. 1:**
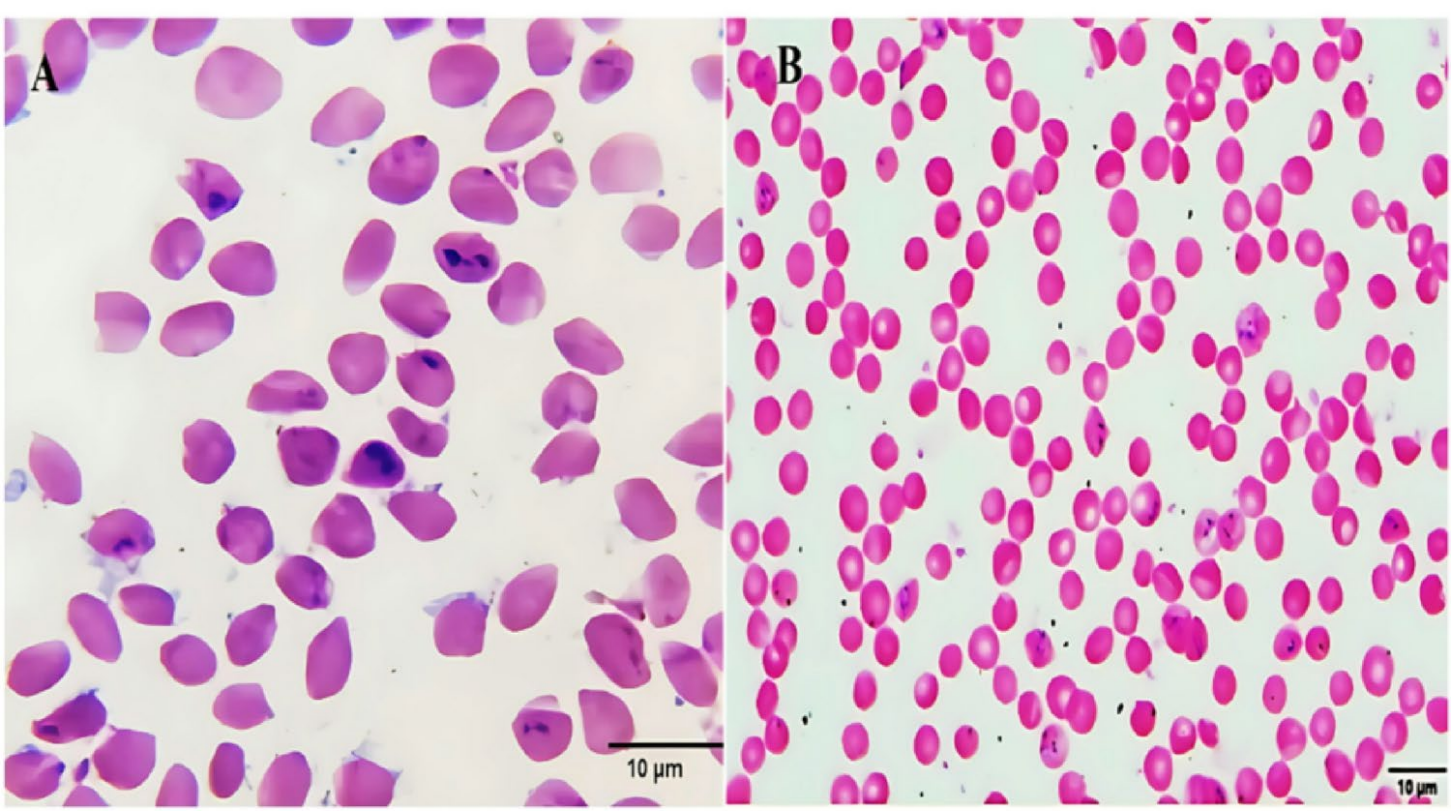
Intraerythrocytic forms of *B. bigemina* in cattle blood smears.** A** Light infection showing few paired pyriform (pear-shaped) and amoeboid forms within red blood cells.** B** Severe infection with numerous parasites observed within erythrocytes. (Giemsa stain; scale bar = 10 μm)

*Babesia* parasites, primarily *Babesia bigemina and Babesia bovis*, were observed within red blood cells. *B. bigemina* was primarily identified as paired pyriform bodies, while *B. bovis* appeared as single forms or irregular pairs (Fig. 1). The intensity of infection varied among cases; in some instances, only a light parasitemia was detected, while in others, the infection was markedly severe, with numerous parasites evident within erythrocytes.

### Prevalence of* Babesia* sp. infection across risk factors

The overall prevalence of *Babesia sp*. infection was determined to be 31.9% (108/339) by microscopy. We reported microscopy-based prevalence as it remains the primary diagnostic standard in many field settings in the region, allowing for direct comparison with historical data. However, the more sensitive molecular methods revealed a higher prevalence, with cPCR detecting *Babesia* DNA in 41.9% (142/339) of samples and LAMPin 40.7% (138/339) Table 1. Seasonal variation significantly impacted infection dynamics. The highest infection rate by microscopy (37.8%) was observed in the summer season, while winter showed a significant drop to 17.0% (RR = 0.45, 95% CI: 0.28–0.99; OR = 0.33, 95% CI: 0.17–0.62; *P* = 0.03). No significant associations were found with gender or age (*P* 0.05) Table 1.

**Table 1:** Prevalence of *Babesia sp.* infection across risk factors

Risk factors		Total	Non_infected	Infected (MO)%	cPCR %	LAMP %	Risk_ratio	Lower_upper	Odd_ratio	Lower_ upper_CI	Chi_square
Season	Summer	119	74	45 (37.82)	48.6	47.2	1	Ref*	1	Ref*	Ref*
	Autumn	100	66	34 (34)	43.7	42.5	0.89	(0.63, 1.29)	0.85	(0.48, 1.48)	0.56
	Winter	45	36	9 (20)	25.7	25	0.53	(0.28, 0.99)	0.42	(0.17, 0.92)	0.03
	Spring	75	55	20 (26.67)	34.3	33.3	0.71	(0.45, 1.09)	0.6	(0.31, 1.12)	0.11
Gender	Male	151	102	49 (32.45)	41.8	40.6	1	Ref*	1	Ref*	Ref*
	Female	188	129	59 (31.38)	40.5	39.2	0.97	(0.71, 1.32)	0.95	(0.60, 1.51)	0.83
Age	< 1	51	33	18 (35.29)	45.4	44.1	1	Ref*	1	Ref*	Ref*
	1–3	100	65	35 (35)	45	43.8	0.99	(0.63, 1.57)	0.99	(0.49, 2.03)	0.97
	3	188	133	55 (29.26)	37.6	36.6	0.83	(0.54, 1.28)	0.76	(0.40, 1.48)	0.41

Ref*: reference value. CI, Confidence Interval. Statistically significant* P*-value < 0.05

### Molecular Diagnosis: LAMP Versus cPCR in Clinically Suspected Cattle

Out of the 20 blood samples collected from clinically suspected cattle, microscopy identified intra-erythrocytic parasites in 5 samples (25%). In contrast, LAMP detected *Babesia* DNA in 14 samples (70%), and cPCR confirmed the presence of *Babesia* DNA in 15 samples (75%). Supplementary Figs. 2 and 3 show LAMP and PCR results, respectively.

**Fig. 2:**
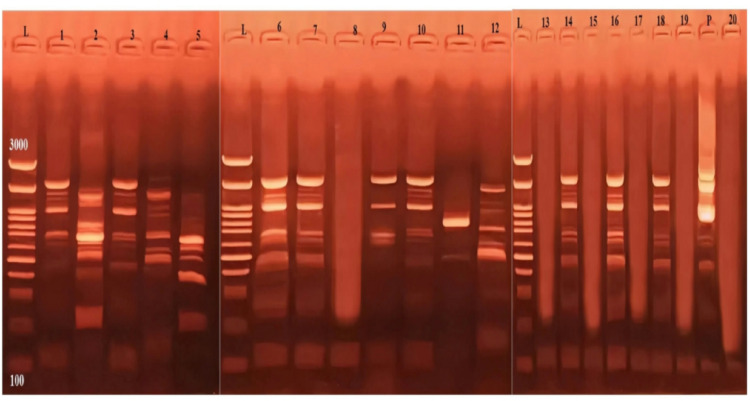
LAMP assay results for B. bigemina and B. bovis. detection. Lane (L) represents the DNA ladder (100-3000 bp), Lane (P) represents the positive sample, while Lane (N) represents the negative sample. Lanes 1 to 7, 9 to 12, 14, 16, 18 show positive LAMP products with a ladder-like pattern, while lanes 8, 13, 15, 17, 19, and 20 are negative

**Fig. 3:**
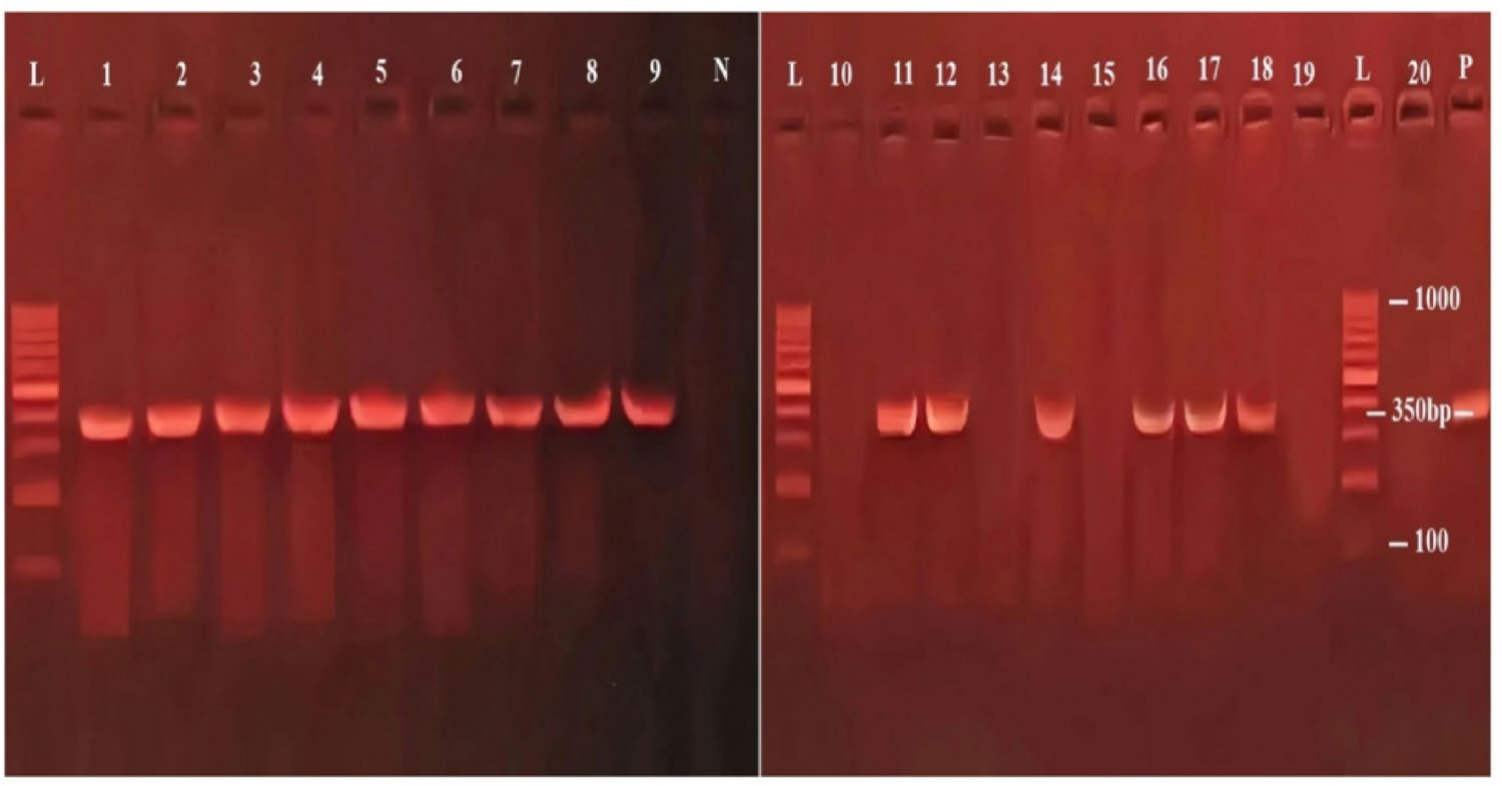
Agarose gel electrophoresis (1.5%) showing PCR amplification products of the B. bigemina and B. bovis. 18S rRNA gene (350 bp). Lane L: DNA size marker, Lane P: positive control, Lane N: negative control. Samples 1-9, 11, 12, 14, 16,17 and 18 are positive, while samples 10, 13, 15,19, and 20 are negative

### Target Pathogen Specificity

In this study, microscopic examination was used for the general detection of *Babesia* parasites within erythrocytes. The molecular assays (LAMP and cPCR) were designed to target the 18 S rRNA gene of *Babesia* sp., allowing for the broad detection of the parasites with the genus. Subsequent species-specific PCR on a subset of positive samples confirmed the presence of both *B. bovis* and *B*. *bigemina* in the study area. While our primer sets are capable of detecting other *Babesia* species, the analysis confirmed that *B. bigemina* and *B. bovis* were the predominant species infecting cattle in this cohort.

### Evaluation of Different Diagnostic Assays Against cPCR as a Reference Test

Using cPCR (142 positive, 197 negative) as the reference standard, microscopy showed Sensitivity 76.06% (108/142 true positive), Specificity: 100% (197/197 true negative), PPV: 100%, and NPV: 85.28%. The low NPV reflects infections detected by molecular methods that were missed by microscopy. Significant discordance was observed (McNemar’s χ^2^ = 34, *p* < 0.001; κ = 0.7869, *p* < 0.001), indicating only substantial agreement with cPCR.In comparison, LAMP exhibited Sensitivity: 97.18% (138/142 true positive), Specificity: 100% (197/197 true negative), PPV: 100%, and NPV: 98.01%. No significant discordance was found (McNemar’s χ^2^ = 2.25, *p* = 0.1336; κ = 0.976, *p* < 0.0001), demonstrating almost perfect agreement with cPCR.(Table 2).

**Table 2:** Classification of animals according signs and comparison of diagnostic performance of blood film and LAMP assays against cPCR

Diagnostic test	Evaluation study											
	Positive (%)	(TP)	(TN)	(FP)	(FN)	Sensitivity (%)	Specificity (%)	(PPV) (%)	(NPV) (%)	(CPV) (%)	Kappa coefficient	p-value
Blood smear	108/339 31.9%	108	197	0	34	76.06	100	100	85.28	92.64	0.7869	1.52
LAMP	138/339 40.7%	138	197	0	4	97.18	100	100	83.30	99	0.9757	0.1

TP, true positive; TN, true negative; FP, false positive; FN, false negative; PPV, positive predictive value; NPV, negative predictive value; CPV, combined predictive value

### Diagnosis Comparison of LAMP Assay and cPCR Against Blood Smear

The diagnostic performance of LAMP and cPCR for detecting Babesia DNA in clinically positive cattle was evaluated using microscopy (108 positive, 231 negative) as the reference standard (Table 3).

**Table 3:** Comparison of LAMP assays and cPCR against blood smear

Diagnostic test	Evaluation study						
	Sensitivity	Specificity (%)	(PPV)	(NPV)	(CPV) (%)	Kappa coefficient	McNemar's test *p*-value
LAMP	97.18	100	100	98.01	99	0.88	0.98
cPCR	100	100	100%	100%	100	0.88	1

PPV, positive predictive value; NPV, negative predictive value; **CPV**, combined predictive value

LAMP showed 97.18% sensitivity and 100% specificity, while cPCR demonstrated 100% sensitivity and 100% specificity. Both assays revealed higher detection rates than microscopy, identifying additional subclinical infections that were missed by the blood smear method. The PPV for LAMP and cPCR were 100%, and the NPVwere 98.01% and 100%, respectively, ensuring no false negatives by cPCR. The Combined Predictive Value CPV reached 99.0% for LAMP and 100% for cPCR.When comparing the diagnostic performances of LAMP and cPCR, the results showed almost perfect agreement, with a Cohen’s kappa coefficient of 0.98 )*P* = 0.13(, indicating a high level of consistency between the two molecular methods. McNemar’s test revealed no significant difference in their diagnostic performance (*p* = 0.13), reinforcing the similarity in their diagnostic accuracy. (Tables 3and 4).

**Table 4:** Comparison of babesiosis prevalence assessed by the three diagnostic techniques used

Diagnostic test	Evaluation study						
	Sensitivity (%)	Specificity (%)	(PPV) (%)	(NPV) (%)	(CPV) (%)	Kappa coefficient	McNemar's test *p*-value
LAMP	100	97.18	98.01	100	99	0.88	0.98
cPCR	100	100	100	100	100	0.88	1

### LAMP Assay for Bovine Babesiosis Detection (Two Visualization Methods as a Field Test)

**Fig. 4:**
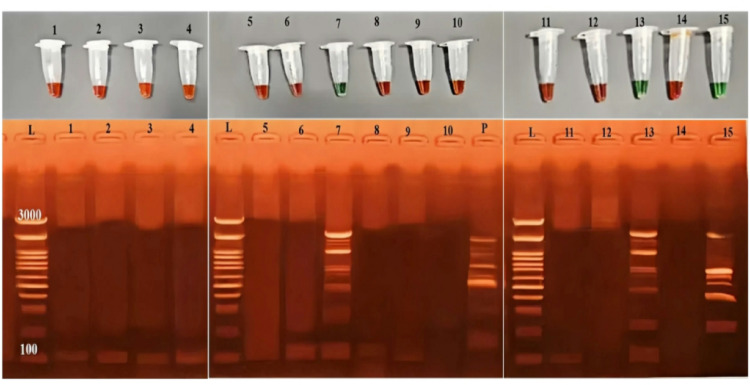
Results of the LAMP test for the detection of bovine babesiosis. The DNA ladder (100–3000 bp) is shown by lane (L), the positive sample by lane (P), and the negative sample by lane (N). The presence of babesiosis DNA is confirmed by positive LAMP products with a ladder-like pattern in lanes 7, 13, and 15. After SYBR Green was added, the positive samples turned green. In other lanes, negative samples remained orange in color

For further assessments, 15 blood samples were selected from cattle exhibiting hemoglobinuria as the sole clinical sign of *Babesia* infection, with blood films previously testing negative for *B. bigemina* and *B. bovis*. The samples underwent LAMP testing to detect *Babesia* DNA. To validate the results, two visualization methods were used: gel electrophoresis and SYBR Green I dye. The results identified three positive samples (7, 13, and 15), as shown in Fig. 4.

## Discussion

While various detection methods have been developed and are used worldwide, many farms, especially in rural regions, still lack access to affordable and practical diagnostic techniques. LAMP has emerged as a rapid, easy-to-perform assay suitable for on-site detection and has been successfully applied for diagnosing parasitic diseases in both humans and animals. This study aims to compare the sensitivity and specificity of LAMP against conventional PCR (cPCR) and microscopy for detecting *Babesia* species in blood samples collected from cattle heavily infested with ticks in Egypt. The results reveal that the overall prevalence of *Babesia sp*. infection detected by microscopy in the current study was 31.9%, while molecular methods revealed significantly higher infection rates (cPCR: 41.9%; LAMP: 40.7%). The microscopy-based prevalence reported in our study appears higher than the 25% reported in Pakistan [22]; however, direct comparisons should be made with caution due to potential methodological differences in detection protocols and varying epidemiological contexts. Nonetheless, these findings underscore the considerable burden of *Babesia* infections in cattle populations exposed to *Rhipicephalus (Boophilus) annulatus* ticks, consistent with its documented status as Egypt's predominant cattle tick species [21, 23].

The observed seasonal infection pattern—peaking in summer (37.82%) and declining to its lowest in winter (20%; OR = 0.42, 95% CI: 0.17–0.92,* P* =  0.03)—closely aligns with known tick population dynamics driven by temperature and humidity variations [24]. These results reinforce the critical importance of implementing targeted tick control strategies during high-risk seasonal periods. While males and young calves (< 1 year) showed marginally higher infection rates, the absence of statistical significance suggests relatively uniform susceptibility across demographic groups within the study population [25]. This demographic neutrality implies that management interventions should prioritize herd-wide approaches rather than targeting specific subgroups.

Clinical manifestations, including high fever, hemoglobinuria, jaundice, and severe weight loss, were consistent with classic presentations of acute babesiosis [26]. The observed correlation between heavy tick infestations and more severe clinical symptoms further supports the paradigm that higher parasitic loads exacerbate disease pathology [27], emphasizing the necessity of integrated tick surveillance programs to mitigate economic impacts.

### Evaluation of Sensitivity

The diagnostic sensitivity and specificity of the assays were evaluated using samples classified as 'true positives' or 'true negatives' based on cPCR results. The analytical sensitivity (limit of detection) for both LAMP and cPCR was determined using serial dilutions of plasmid DNA containing the target gene fragment, confirming the high analytical sensitivity of both molecular methods (in future studies).

Key diagnostic insights emerged from our evaluation, demonstrating that microscopic diagnosis of babesiosis has substantial limitations, characterized by low sensitivity (76.64%), and identifying only 25% of clinically suspected cases, aligning with its well-documented inadequacy in low-parasitemia and subclinical infections [8, 20]. LAMP examination demonstrated near-perfect agreement with cPCR (κ = 0.875), achieving 97.18% sensitivity and 100% specificity. More importantly, the clinical utility of this technique was particularly evident in detecting hemoglobinuria-positive cases overlooked by microscopy [12], highlighting its value for early infection diagnosis. The field adaptability of LAMP—enabled by equipment-free visual interpretation using SYBR Green I and rapid isothermal amplification [10, 28, 29]—positions it as a viable alternative to cPCR for frontline, on-site surveillance in resource-constrained settings.

### Comparison of Test Performance

A notable difference in detection rates was observed between field samples and those from clinically suspected cattle. In the general field samples, cPCR showed a slightly higher detection rate (41.9%) compared to LAMP(40.7%), consistent with studies reporting LAMP's superior sensitivity in detecting low-level parasitemias. In contrast, in clinically suspected animals with likely higher parasitic loads, both methods performed robustly, with cPCR detecting a marginally higher rate (41.9%) than LAMP (40.7%) in this small sample set. This minor discrepancy may be attributed to natural variations in DNA extraction efficiency or PCR inhibition in individual samples.

Results from comparing the two molecular methods used in this study revealed that both assays demonstrated perfect sensitivity (100%) but lower specificity against microscopy (LAMP: 97.18%; cPCR: 100%), primarily attributable to microscopy's high false-negative rate rather than false positives by molecular assays. LAMP's operational advantages—including rapid turnaround (40 min), minimal equipment requirements, and cost-effectiveness [30]—make it preferable for endemic regions, though cPCR retains value for reference confirmation.

Given that hemoglobinuria is a recognized early clinical sign of *Babesia* infection in cattle, animals exhibiting this symptom but testing negative on conventional Giemsa-stained blood smears were further evaluated using LAMP [12]. LAMP successfully detected *Babesia* DNA in samples initially overlooked by microscopy, highlighting its superior sensitivity, particularly in cases of low parasitemia or early infection. Its simplicity, rapid turnaround, and adaptability to field conditions make LAMP a valuable tool for routine diagnostics in endemic areas. Additionally, SYBR Green I dye served as an effective and straightforward visual indicator for confirmatory purposes, with positive samples turning green and negative samples remaining orange. This dual detection strategy, combining gel electrophoresis with SYBR Green I visualization, enhanced the reliability and practicality of LAMP-based diagnosis for *Babesia* spp. [29].

Recent advances in quantitative real-time PCR (qPCR) have significantly improved the detection and quantification of *Babesia* infections in cattle, offering higher sensitivity and specificity compared to traditional microscopy and conventional PCR methods. TaqMan-based assays targeting conserved genes (18S rRNA, *msa*, and mitochondrial sequences) enable rapid and reliable differentiation of *B. bovis* and *B. bigemina*, while SYBR Green and multiplex qPCRs allow cost-effective and simultaneous detection of multiple haemoparasites. These assays have been validated for analytical and diagnostic performance, demonstrating detection limits as low as a few parasite copies per reaction and excellent specificity under field conditions [31–33]. Consequently, qPCR-based techniques have become indispensable tools for epidemiological surveillance, carrier detection, and control programs of bovine babesiosis worldwide.

*Limitation related to molecular confirmation* Although the current study employed species-specific primer sets to detect *Babesia bovis* and *B. bigemina*, sequencing of PCR amplicons was not performed to validate the molecular findings. The absence of sequencing confirmation represents a limitation, as it prevents definitive verification of the amplified products and excludes the possibility of non-specific amplification or the presence of closely related *Babesia* species. Moreover, while microscopy was used for initial screening, this method has limited sensitivity in carrier or chronically infected animals with low parasitemia, potentially leading to under- or misdiagnosis. Therefore, future investigations should incorporate sequencing and phylogenetic analysis of PCR products to confirm species identity and improve the accuracy of *Babesia* detection and epidemiological assessments.

## Conclusions

While microscopic examination remains useful for detecting acute high-parasitemia cases, molecular tools like LAMP are warranted for comprehensive surveillance programs. The ability of LAMP to detect subclinical and early-stage infections with sensitivity and specificity comparable to cPCR represents a significant advancement in babesiosis management, particularly in endemic regions with limited diagnostic infrastructure. Future research should validate these findings in larger sample sizes across broader geographical areas and characterize the genetic diversity of circulating *Babesia* strains to optimize control strategies.

## Data Availability

No datasets were generated or analysed during the current study. The authors declare no conflict of interest.

## Abbreviations

B. bovis: *Babesia bovis*
B.bigemina: *Babesia bigemina*
MO: Microscopic observations
CI: Confidence interval
cPCR: Conventional polymerase chain reaction
CPV: Combined predictive value
DNA: Deoxyribonucleic acid
EDTA: Ethylenediaminetetraacetic acid
ELISA: Enzyme-linked immunosorbent assay
FN: False negative
FP: False positive
TN: True negative
TP: True positive
ICT: Immunochromatographic test
IFAT: Immunofluorescent antibody test
LAMP: Loop-mediated isothermal amplification
NPV: Negative predictive value
OR: Odds ratio
PCR: Polymerase chain reaction
PPV: Positive predictive value
RR: Risk ratio
SYBR Green I: A fluorescent nucleic acid stain
